# Postoperative hip center position is associated with gait symmetry in range of axial rotation in dysplasia patients after THA

**DOI:** 10.3389/fsurg.2023.1135327

**Published:** 2023-05-10

**Authors:** Yi Hu, Diyang Zou, Mengda Jiang, Qingyu Qian, Huiwu Li, Tsung-Yuan Tsai, Jingwei Zhang

**Affiliations:** ^1^ Shanghai Key Laboratory of Orthopaedic Implants, Department of Orthopaedic Surgery, Shanghai Ninth People's Hospital, Shanghai Jiao Tong University School of Medicine, Shanghai, China; ^2^ School of Biomedical Engineering, Med-X Research Institute, Shanghai Jiao Tong University, Shanghai, China; ^3^ Department of Radiology, Shanghai Ninth People's Hospital, Shanghai Jiao Tong University School of Medicine, Shanghai, China; ^4^ TaoImage Medical Technologies Corporation, Shanghai, China

**Keywords:** total hip arthroplasty, gait symmetry, fluoroscope, hip center, developmental hip dysplasia (DDH)

## Abstract

**Background:**

This study aimed to explore whether pre- or postoperative hip structures or surgical changes significantly influence hip range of motion (ROM) symmetry in patients with hip dysplasia during gait after total hip arthroplasty (THA) and provide possible surgical suggestions.

**Methods:**

Fourteen patients with unilateral hip dysplasia underwent computed tomography before and after surgery to create three-dimensional hip models. Pre- and postoperative acetabular and femoral orientations, hip rotation centers (HRC), and femoral lengths were measured. Bilateral hip ROM during level walking after THA was quantified using dual fluoroscopy. The ROM symmetry in flexion-extension, adduction-abduction, and axial rotation was calculated using the symmetry index (SI). The relationship between SI and the above anatomical parameters and demographic characteristics was tested using Pearson's correlation and linear regression.

**Results:**

The average SI values for flexion-extension, adduction-abduction, and axial rotation during gait were −0.29, −0.30, and −0.10, respectively. Significant correlations were detected mainly in the postoperative HRC position. A distally placed HRC was associated with increased SI values for adduction-abduction (*R* = −0.47, *p* = 0.045), while a medially placed HRC was associated with decreased SI values for axial rotation (*R* = 0.63, *p* = 0.007). A regression analysis indicated that horizontal HRC positions significantly determined axial rotational symmetry (*R*^2^ = 0.40, *p* = 0.015). Normal axial rotation SI values were achieved with HRC between 17 mm medially and 16 mm laterally.

**Conclusions:**

Postoperative HRC position was significantly correlated with gait symmetry in the frontal and transverse planes in patients with unilateral hip dysplasia after THA. Surgical reconstruction of the HRC to between 17 mm medially and 16 mm laterally may contribute to gait symmetry.

## Introduction

Developmental dysplasia of the hip (DDH), a leading precursor of secondary osteoarthritis (OA), is seen in 20%–40% of patients with hip OA (1). This may eventually lead to total hip arthroplasty (THA) to relieve pain and improve function (1). However, a broad range of pathomorphological changes from the acetabular and femoral sides, including bony acetabular defects and a high-riding or even dislocated femoral head, make THA in patients with DDH a highly complex reconstruction (2). In addition, the incidence of postoperative limping in such patients increases with deformities, reaching 45.5%–83.0% in the Crowe III–IV type (3–5). Other complication risks, including aseptic loosening, dislocation, and polyethylene wear, are much higher than in these patients than in those with primary OA (6–8).

Because of pain and weakness, lingering limping or asymmetrical gait patterns are common in patients after THA (9). However, despite proper rehabilitation, such asymmetry usually persists for more than 12 months after surgery (10). This not only impairs life quality but might increase joint loading and OA risks in the contralateral limb and induce further prosthetic failure on the affected side (11, 12). Therefore, optimizing postoperative gait patterns and avoiding persistent asymmetry are crucial for improving clinical outcomes.

Anatomical hip reconstruction is required in standard THA to ensure biomechanical superiority and normal walking mechanics (7). However, due to the degree of deformity, reconstruction goals may not be all attainable in patients with DDH, which has a far-reaching influence on gait patterns (4, 13, 14). Although bringing the hip center back down to the true acetabulum makes hip rotating on the same level with the unaffected side, and favors gait symmetry, cup coverage is always insufficient due to acetabular defects in patients with DDH, and surgeons sometimes have to establish the center at a slightly superior position (15). The restoration of equal leg length contributes to gait symmetry, whereas in DDH patients with subluxated or high-riding hip centers, a deeper stem fixation or femoral shortening osteotomy may be necessary to reduce the hip to avoid potential neurovascular injury (16). Applying a conventional conical prosthesis is sufficient for appropriate joint alignment and anatomical restoration of the femoral offset (FO), which guarantees effective abductor action in primary OA patients (17). However, in DDH patients with variant acetabular or femoral angles and contracted abductors, modular components are sometimes selected to individually adjust joint alignments and additional increase FO to enhance abductor strength and avoid limping. When anatomical reconstruction goals become challenging and cannot be achieved in THA for patients with DDH, strategies that should be adopted to achieve better gait symmetry deserve further investigation. Should surgeons chase anatomic reconstruction despite pathomorphology or avoid immense surgical alterations and yield to preoperative deformities? Besides, which exact hip structure among several reconstruction goals should be anatomically rebuilt with priority, and which hip structure and to what extent could be compromised during operation also needs to be clarified.

Different reconstruction strategies during THA for patients with DDH result in different postoperative hip biomechanical environments and further affect gait symmetry and clinical outcomes (18–20). Understanding how reconstruction strategies influence postoperative gait patterns is crucial for making necessary adjustments. Previous studies primarily measured hip structures using plain radiographs and investigated their relationship with skin marker-based gait analysis. Karaismailoglu et al. explored the effect of hip center height using gait analysis. They found that a unilateral 15-mm superiorly placed hip center on x-ray reduced hip motion range on the operated side and increased fall risks, while such an effect disappeared when patients' bilateral hips were symmetrically reconstructed at the same height (20, 21). When the leg length discrepancy is within 20 mm on radiographs, patients with unilateral DDH showed less gait symmetry than healthy controls at the 5-year follow-up after THA (22). Greater bilateral symmetry was detected if the leg length discrepancy was less than 10 mm (22). Sariali et al. performed gait analyses of patients after THA. They found that an isolated decrease of 15% in FO induced statistically significant gait asymmetry between the sides with a reduced range of motion and a lower maximal swing speed on the operated side, whereas a restored or increased FO did not lead to gait symmetry alterations (23). However, to the best of our knowledge, there are limited quantitative data on the association between precisely measured hip structures and gait characteristics in patients with DDH after THA.

To identify potential factors influencing gait symmetry, here we first used a dual fluoroscopic imaging system (DFIS) based on three-dimensionally (3D) computed tomography (CT) modeling to measure the *in vivo* six degrees of freedom (6-DOF) hip kinematics of patients with DDH after THA during gait. We then tested the correlation between the characteristics of the gait symmetry index (SI) and the patients' demography and hip anatomic structures before and after THA and their changes during surgery. We aimed to determine the following: (1) the relationship between the above parameters and gait SI in such DDH patients; (2) whether demographic information, pre- or postoperative anatomic structures, or changes during surgery had the most significant influence on gait SI and should be managed carefully during THA; and (3) provide specific reconstruction suggestions that most benefit the recovery of gait symmetry in DDH patients following THA. We hypothesized that postoperative hip structure would be the most influential factor affecting gait symmetry after THA.

## Materials and methods

### Patient demographics

This retrospective study was approved by the institutional review board. Written informed consent was obtained from each participant before the study. Fourteen patients (three men, 11 women) with excellent functional unilateral THA (Harris hip score, > 90 points) for end-stage hip OA secondary to DDH on one side and a healthy status on the other side based on radiological findings were recruited. Patients bilaterally affected, with diseases affecting joint movements, or with a history of other hip surgeries or THA complications such as dislocation, subluxation, or periprosthetic fractures were excluded. Patients who underwent THA for less than 1 year were also excluded. Five patients were diagnosed with Crowe type II, four with Crowe type III, and the other five with Crowe type IV DDH (2). The average follow-up period was 3.4 years (±1.0; range, 2.0– 5.4; Table 1).

**Table 1:** Patient demographic characteristics.

Characteristic	Value
Sex (male/female)	3/11
Age (y)	57.2 ± 12.4
Height (m)	1.62 ± 0.06
Weight (kg)	61.7 ± 8.7
BMI (kg/m^2^)	23.3 ± 2.3
Follow-up (y)	3.4 ± 1.0
Crowe classification (type I/II/III/IV)	0/5/4/5

Values are shown as number or mean ± standard deviation.

### Surgical technique and rehabilitation

All THA were performed by the same group of qualified surgeons (HWL and JWZ) using a posterolateral approach. Generally, the acetabular cup is implanted using a press-fit technique in an anatomically or slightly superior place. The femoral component that best matched the broached intramedullary canal was selected. In highly dislocated cases, the capsule was cut off for reduction. Muscle and soft tissue release was performed as less as possible, and no release was made of the gluteus maximus or iliopsoas. None of the patients underwent femoral shortening osteotomy. Cementless cups (SecurFit, Stryker, USA; Pinnacle, DePuy, USA) and stems (SecurFit, Stryker; Corail, DePuy; and S-rom, DePuy) were implanted in all patients.

The patients began passive range of movement exercises 24 h postoperative and mobilized non-weight-bearing in the first week postoperative. Partial weight-bearing as tolerated was allowed for the following 6 weeks. Thereafter, progressive weight-bearing with crutches was performed, with unrestricted walking allowed after 8 or 12 weeks.

### CT-Based 3d modeling and anatomical parameter measurements

Each patient underwent CT (SOMATOM Definition Flash, Siemens, Germany) covering the bilateral anterior superior iliac spine and posterior borders of the medial and lateral condyles with a 0.5-mm interspace thickness before and after THA. The CT images were imported into Amira (Amira 6.7; Thermo Fisher Scientific, Rockford, IL, USA) to create 3D surface models of the hip using a region-growing method. Models of the pelvic and bilateral femurs before and after THA and the implanted prosthesis were prepared.

**Figure 1:**
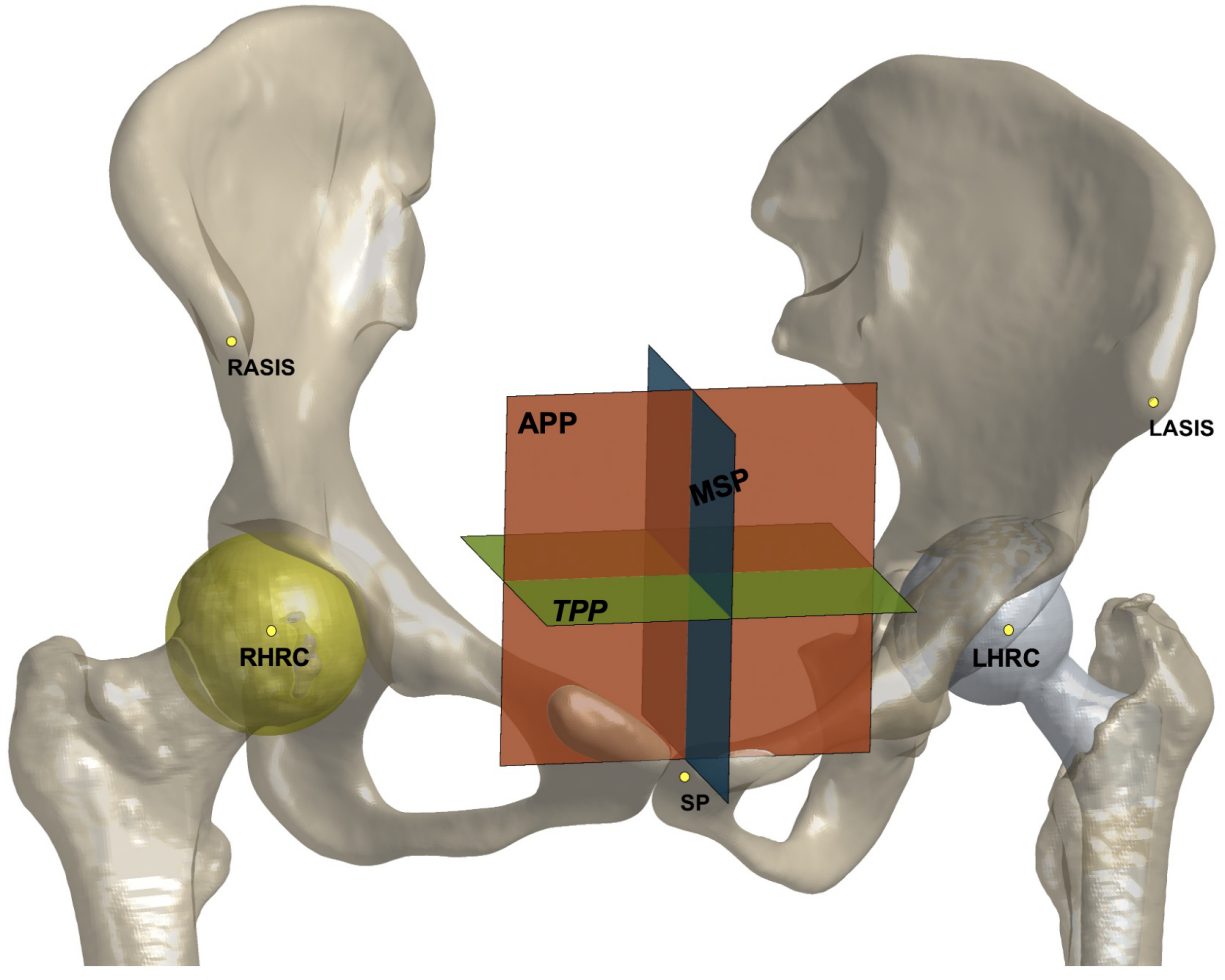
The anterior pelvic plane (APP), transverse pelvic plane (TPP), medial sagittal plane (MSP), and hip rotation center (HRC) shown on models of the pelvis and implants. The relative distance to three plans was recorded as HRC position.

The above models were imported into Rhino 5.0 (Rhinoceros, Robert McNeel & Associates, Seattle, WA, USA) for anatomical measurements. The anterior pelvic plane (APP), established by the bilateral anterior superior iliac spine (ASIS) and symphysis pubis (SP), was considered the reference plane for the anatomical component position. The transverse pelvic plane (TPP) was perpendicular to the APP and horizontal to the connection between the bilateral ASIS. The medial sagittal plane (MSP) was perpendicular to the APP and TPP and passed through the midpoint of the SP. The center of the best-fit sphere to the femoral head was defined as the center of hip rotation center (HRC). The relative distance of the HRC to the APP, TPP, and MSP compared to the healthy side was recorded as the HRC position, with positive values indicating a more proximally, laterally, or anteriorly placed HRC (Figure 1). When the pelvis is seriously asymmetrical, the ASIS on the healthy side and its mirroring are alternatively used to establish an APP (24). Acetabular anteversion and inclination were measured according to Murray's anatomical definitions (25). Femoral anteversion was defined as the angle formed by the femoral neck axis, the plane passing through the posterior medial and lateral femoral condyles, and the lesser trochanter. The combined anteversion was calculated as the sum of the acetabular and femoral anteversions (26). The absolute femoral length was measured as the distance between the HRC and the midpoint of the femoral condyles and the relative values between the sides were recorded. Positive values indicated a prolonged affected femur. The values of these variables before and after surgery and the changes in THA were collected for further testing.

### Hip kinematics measurements during gait and symmetry Index

The DFIS was first established by placing two mobile fluoroscopes (ARCADIS Varic, Siemens, Germany) in nearly orthogonal positions. Each patient then performed level walking on a treadmill at a speed of 1 km/h under DFIS surveillance (30 snapshots/s with an 8 ms pulse width, 60–80 kV, and 0.042–0.066 mAs) for the implanted and contralateral healthy hips. Next, the 3D hip models were imported into MATLAB (2020a; MathWorks, Natick, MA, USA), in which the pelvic and femoral local coordinate system was defined according to International Society of Biomechanics recommendations (27). Subsequently, a series of fluoroscopic images and hip models was imported into a simulated environment using MATLAB. The hip models were translated and rotated through 6-DOF in this 3D virtual space until the models matched the fluoroscopic images. The tracking errors for this DFIS technique are <0.35 mm and <0.55° (28). The maximum range of motion (ROM) in flexion-extension, adduction-abduction, and axial rotation was calculated for the implanted and contralateral healthy hips.

Gait asymmetries in this study were quantified using the SI, a validated parameter that summarizes overall gait kinematic quality (29). Several spatiotemporal parameters of the gait cycle, including joint angles and velocities, can be calculated for SI scores (11, 30–32). In this study, we selected the ROM for calculation consistent with previous studies (32). The SI was calculated as follows:

$$SI=\frac{X_{op}-X_{he}}{0.5\;\times \;(X_{op}+X_{he})}$$

where X_op_ is the ROM of the operated side and X_he_ is the ROM of the contralateral healthy side. The SI values for flexion-extension, adduction-abduction, and axial rotation for each patient were calculated. To define normative symmetry values, a 95% confidence interval (CI) was calculated for each SI in the normal THA group. The results by Tsai et al. were referred to as normal after the elimination of extreme values, and the calculated SI in 95% CI were −0.42 to 0.20 for flexion-extension in the sagittal plane, −0.66 to 0.80 for adduction-abduction in the coronal plane, and −0.75 to 0.87 for axial rotation in the transverse plane.

### Statistical analysis

All statistical analyses were performed using SPSS Statistics for Mac (version 26.0; IBM Corp., Armonk, NY, USA). We primarily focused on the most influencing variable on gait symmetry, and further explored the relationship between them and possible surgical suggestions. We first used Pearson's correlation analysis to test the relationship between various linear variables and gait SI in the three directions. These variables included demographic characteristics, preoperative and postoperative component positions, and surgical changes. Then, three forward stepwise multiple regression models were established to determine the linear variables that contributed the most to the SI values in each direction. The level of significance was set at *p* < 0.05.

## Results

### Postoperative gait SI values and Hip anatomic parameters

On average, the SI value for flexion-extension in these patients was −0.29 ± 0.36, close to that for adduction-abduction of −0.30 ± 0.37. More deviated SI values of −0.10 ± 0.53 were achieved with the axial rotation (Table 2). The average SI values in the three directions were all negative, indicating that the implanted side had a smaller ROM than the healthy side, and there was restriction to some extent. Specifically, eight patients had normal SI values for flexion-extension and 12 patients for adduction-abduction and axial rotation (Table 2). Only six (43%) patients had normal SI values in all three directions.

**Table 2:** Postoperative SI values in three directions during gait.

Patient ID	Flexion-extension	Adduction-abduction	Axial rotation
1	−0.26	−0.34	0.27
2	−0.08	−0.55	−0.58
3	0.05	−0.03	0.71
4	−0.47	0.40	−0.74
5	−1.01	−0.41	−0.14
6	−0.38	−0.52	0.45
7	−0.21	0.06	−0.09
8	−0.41	−0.19	0.61
9	−0.44	−0.29	0.09
10	0.14	−0.90	−0.85
11	−0.51	−0.37	0.26
12	−0.75	−0.63	−0.19
13	0.34	0.24	−0.80
14	−0.06	−0.72	−0.47
Mean	−0.29	−0.30	−0.10
SD	0.36	0.37	0.53

SI, symmetry index; SD, standard deviation.

Positive degrees indicate that the operated side moved more than the healthy side and vice versa.

For joint alignments, only average cup anteversion was increased by 0.8 ± 9.8°, with cup inclination, femoral and combined anteversion decreased by 23.4 ± 8.0°, 5.0 ± 11.4° and 4.3 ± 14.8°, respectively (Table 3). THA removed HRC to the distal, medial and posterior direction when compared to the preoperative position, with an average translation of 10.2 mm, 20.8 mm and 3.2 mm, respectively. The postoperative HRC was more superior, medial, and posterior than the contralateral side, with an average translation of 2.0 mm, 4.1 mm, and 2.4 mm. The postoperative femur length discrepancy was 2.0 mm on average, ranging from −9.9 to 10.4 mm (Table 3).

**Table 3:** Hip anatomic parameters before and after THA and their changes during surgery.

	Acetabulum ante. (°)			Acetabulum inc. (°)			Femoral ante. (°)			Combined ante. (°)			HRC P/D* (mm)			HRC A/P* (mm)			HRC M/l* (mm)			Femoral length* (mm)		
	Pre	Post	D	Pre	Post	D	Pre	Post	D	Pre	Post	D	Pre	Post	D	Pre	Post	D	Pre	Post	D	Pre	Post	D
Max.	38.6	45.1	16.5	79.1	51.0	−11.3	51.2	41.9	10.9	89.9	64.6	19.1	53.8	19.7	6.8	22.7	20.6	2.0	35.9	6.7	−9.2	7.3	10.4	24.5
Min.	11.8	10.5	−19.0	56.7	39.4	−39.7	8.4	7.5	−25.5	32.0	33.2	−34.4	−13.9	−23.9	−38.8	−25.1	−29.3	−8.9	5.7	−16.9	−37.8	−30.9	−9.9	−13.7
Avg.	25.4	26.2	0.8	68.5	45.1	−23.4	29.4	24.3	−5.0	54.8	50.5	−4.3	12.1	2.0	−10.2	0.8	−2.4	−3.2	16.7	−4.1	−20.8	−5.2	2.0	4.1
SD	8.3	9.2	9.8	7.2	4.1	8.0	12.6	9.4	11.4	15.1	9.0	14.8	18.1	11.8	14.3	12.7	12.7	3.6	9.5	7.0	8.3	10.4	6.5	9.7

*Relative data compared to the healthy side were used; Ante, anteversion; A/P, anterior/posterior; D, difference calculated by postoperative minus preoperative values; HRC, hip rotation center; Inc., inclination; M/l, medial/Lateral; P/D, proximal/distal; Post, postoperative; Pre, preoperative.

These parameters shown are maximum (Max.), minimum (Min.), average (Avg.) and standard deviation (SD).

### Correlation relationship between Various variables and gait Si

**Figure 2:**
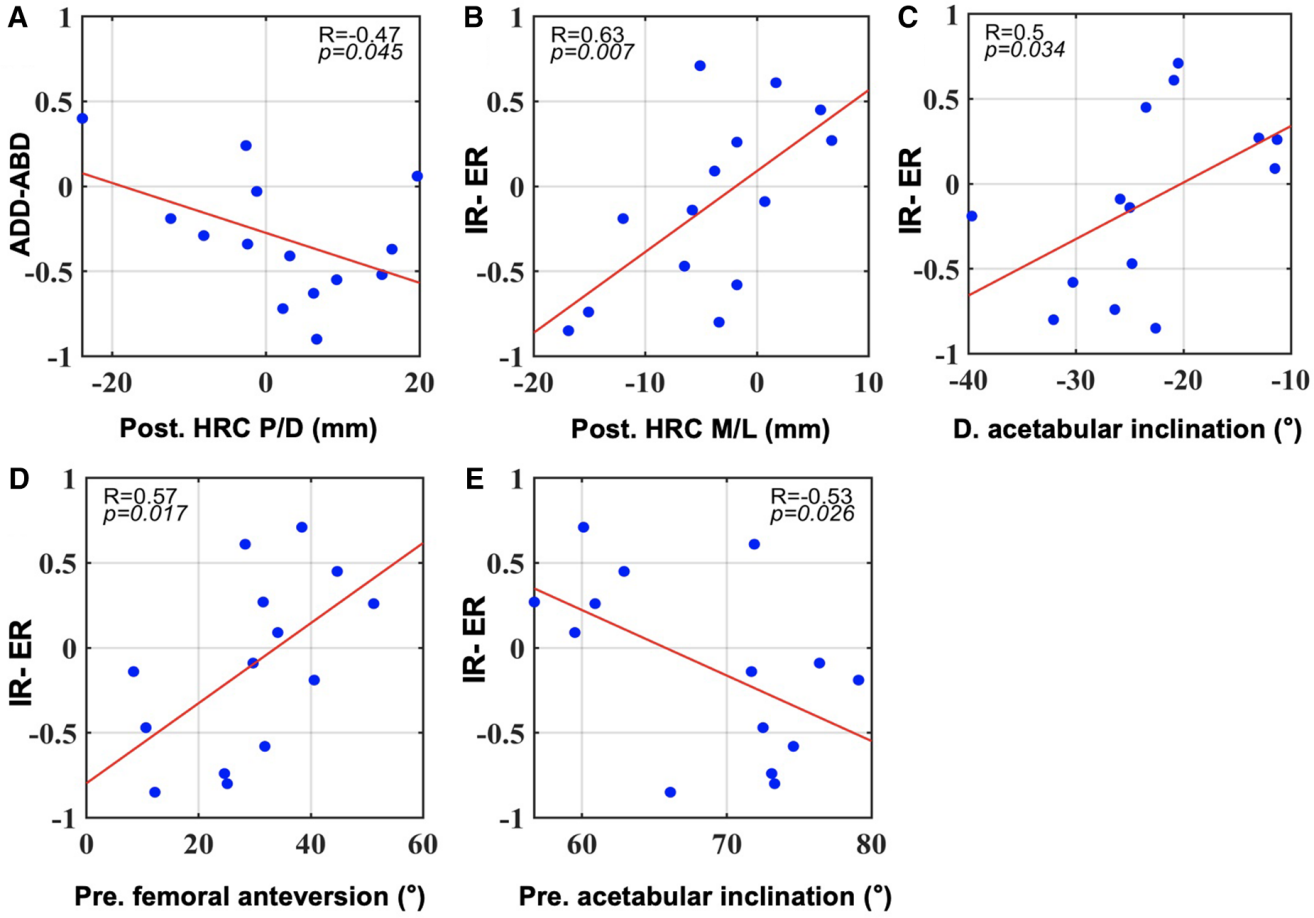
All significant correlations between SI values during gait and pre- and postoperative component positions as well as their changes in surgery (*p* < 0.05). (**A**): correlations with SI for adduction-abduction. (**B–E**): correlations with SI for internal and external rotation. Post, postoperative; Pre, preoperative. (**D**) difference of postoperative minus preoperative data.

Pearson's correlation coefficient analysis revealed significant correlations with gait SI values, mainly in the postoperative joint structures (Figure 2). A superior HRC after surgery correlated with smaller SI values for add-abduction (*R* = −0.47, *p* = 0.045; Figure 2A). Statistically significant positive correlations with SI values for axial rotation were detected in postoperative HRC positions in the M/L direction, differences in acetabular inclination, and preoperative femoral anteversion (Figures 2B–D), and a negative correlation with preoperative acetabular inclination (Figure 2E). There was no significant correlation between the SI values and flexion-extension.

### Specific contributing factors and reconstruction suggestions

A further mathematical model using forward stepwise multiple regression selected the postoperative HRC position in the M/L direction as a significant contributing factor affecting the SI values for axial rotation (Table 4). A laterally placed acetabular cup significantly explained 40% of the increase in SI values for axial rotation (*p* = 0.015; Table 4). Based on the regression model, when the acetabular cup was medially reconstructed by 2 mm, the SI was 0, indicating that both hips had the same ROM in axial rotation. Normal SI values were achieved when the cup was reconstructed between 17 mm medially and 16 mm laterally, probably indicating symmetrical gait patterns in the transverse plane. No demographic or other variables were shown in any of the correlation or regression models. Normality tests of the residuals in the above analyses were all passed.

**Table 4:** Predictors in regression model for axial rotation SI values.

Predictor	Coefficient	*R* ^2^	*P*
Constant	0.09		
Postoperative HRC M/l distance	0.05	0.40	0.015

HRC, hip rotation center; M/l, medial/lateral; SI, symmetry index.

## Discussion

The current study investigated *in vivo* gait symmetry in patients with DDH following unilateral THA during level walking and its correlation with surgical interventions and corresponding reconstruction suggestions. This study found that only 43% of these patients had symmetric gait. Further analysis revealed significant correlations between gait symmetry and postoperative joint structures, and the HRC position was a significant contributor to axial symmetry. In particular, postoperative hip center positions in the M/L direction determined gait axial symmetry. With the hip centers between 17 mm medially and 16 mm laterally, the gait symmetry in axial rotation could probably be improved.

Restoring anatomical hip structures is a general requirement for THA to improve hip biomechanics, support normal gait mechanics, and guarantee satisfactory clinical outcomes (33). However, this is not always possible in some patients due of preoperative deformities. Adjustive and compromised reconstruction strategies are adopted to avoid extreme surgical changes, including femoral shortening osteotomy and high hip center displacement, which contribute to smooth surgery and fast postoperative recovery (34–36). However, such compromised strategies inevitably change hip biomechanics and may alter gait patterns, further affecting clinical outcomes. Previous studies primarily measured hip structures based on plain radiographs or CT scans and investigated the corresponding hip kinematic changes with gait analysis (20–22, 37). These studies had great achievements. However, x-rays are associated with image magnification, and the accuracy of the skin marker-based measurement is limited to soft tissue artifacts, which barely quantify small joint motions such as axial rotation or add-abduction (28). In this study, we used the DFIS tracking technique to quantify *in vivo* hip kinematics with a precision of 0.35 mm and 0.55° (28). We also measured CT-based hip structures. The measurement accuracy was greatly improved in this study, and the findings could be helpful supplements for future studies.

The preoperative hip state probably affected postoperative gait patterns. Foucher et al. found that preoperative gait, HHS, and passive ROM could predict up to 33% variability in postoperative gait (38). A greater preoperative abductor strength is associated with greater postoperative adduction and external rotation moments (38). Preoperative gait and HHS could also predict THA clinical response with a sensitivity of 71.4% and a specificity of 99.1% (39). In our study, preoperative joint alignment was associated with rotational hip symmetry during gait. In this study, we focused on the hip anatomical structures that would be reconstructed in THA and their predictive ability for postoperative gait symmetry. Since preoperative gait or Harris Scores in Foucher's studies were reflections of the overall hip function, our study supplemented the results in terms of surgery and was not contradictory to theirs. Although more significant correlations were detected in the postoperative hip structures in our study, the connection between preoperative hip state and postoperative gait patterns was reasonable. Admittedly, postoperative gait kinematics and symmetry in patients with DDH after THA are complex. Further studies are required to confirm this hypothesis.

The postoperative hip center or cup position is among the most influential factors in hip kinematics and function. Karaismailoglu et al. found that unilaterally elevated hip centers induced a lower extension range of the affected side in patients with DDH, and this phenomenon disappeared with bilateral hip centers of the same height (20, 21). Berninger et al. further found that DDH patients with more proximal cups (> 75 mm from the inter-teardrop line) demonstrated significantly poorer scores in lower extremity function and daily activity than those with more distal cups (40). From a biomechanical perspective, laterally or proximally placed hip centers would increase hip loads and the risk of loosening and should be avoided (41–43). As for gait symmetry, Nie et al. found that cup superior displacement within 12 mm contributes to 3-plane symmetry gait patterns in DDH patients after unilateral THA (44). However, contradictory findings were reported by Leijendekkers et al. that even if the anatomical centers of rotation were reconstructed, modest asymmetries in lower limb kinematics and kinetics were still present in patients with unilateral DDH patients during gait (45). In our study, significant Pearson's correlations were detected between HRC positions and gait adduction-abduction (Figure 2A) and rotational symmetry (Figure 2B). This indicates that postoperative rotation center positions were significant contributors to gait symmetry, particularly in the frontal and transverse planes, and should be treated with caution during THA.

Elevated hip centers have been reported to lead to a less extended ROM of the affected side in patients during gait (20). A similar negative regression relationship between center height and extension range was also observed by Hu et al. (46). While in our study, different results were found that a distally placed HRC was associated with increased SI values for adduction-abduction in the frontal plane. No influence of hip center height on extension or flexion was found. Such discrepancy could be possibly explained by the fact that the aforementioned studies tested flexion and extension separately. While in our study, we calculated the ROM in the sagittal plane, namely flexion and extension, as a whole. Since the extension degrees were far less than the flexion degrees, the connection between the center position and the extension degrees might be overlapped. Delp et al. used computer simulation and found that superior displacement of the cup by 2 cm adversely affected the abductor muscles by decreasing their moment arms (47). Thus, it became reasonable that patients with elevated hip centers might suffer from decreased abductor function, which probably led to abduction restriction, and less adduction-abduction symmetry in the frontal plane. This could possibly explain our results. However, further studies are required to confirm this hypothesis.

In this study, Pearson's correlation and multiple regression analyses identified the hip center position in the M/l direction as the significant predictor of gait rotational symmetry on the transverse plane. With medially placed hip centers, the ROM of axial rotation would probably decrease on the affected side and increase on the unaffected side. A recent study also reported a regression model of hip center M/l displacements and axial rotation degrees, in which medially placed hip centers decreased the internal rotation range of the affected hip (46). Besides, with medialized hip centers, the external rotation of the affected side increased (46). However, the total axial rotation degrees were not calculated, and the authors focused on the motion of only one side, and did not report the other side (46). Compensatory motion adjustments might happen under changed HRC positions. Thus, when considering gait symmetry, bilateral hip movements were both important. The specific link between hip center position and axial rotation requires further study. Moreover, based on the regression model used in this study, normal axial rotation SI values were achieved when the hip centers were between 17 mm medially and 16 mm laterally, suggesting symmetrical axial rotation patterns. Foucher et al. compared responders and non-responders to THA surgery and found a significantly lower peak external rotation moment in non-responders by gait analysis (39). This suggests a subtle role of transverse plane symmetry in promoting a better THA response. Our reconstruction suggestion for hip centers between 17 mm medially and 16 mm laterally could be a beneficial supplement for better transverse plane biomechanical balance when treating patients with DDH and adjusted centers. An inappropriate hip rotation gait could also be attributed to muscle impairments, including dysfunction of the hip abductor lever arm, tightness of the hip adductors, flexors, hamstrings, or gluteus medius, an imbalance between hip rotators, and overactivity of the calf muscles (48). However, further kinetic studies are required to confirm this hypothesis.

Anatomical hip reconstruction and concomitant leg-length equalization are not always easy in patients with severe unilateral DDH. When the cup is implanted superiorly to ensure better host bone coverage, surgeons may adjust the stem depth to achieve an equal leg length. In contrast, when the cup is placed in the anatomical acetabulum, deeper stem fixation or even femoral osteotomy may be required to smoothly reduce the hip. Whether cup position or leg length has greater influence on hip biomechanics remains controversial. However, no factors involving femoral length were identified in any correlation or regression analyses of this study; rather, only the vertical HRC positions correlated with hip adduction-abduction symmetry (Figure 2A). This finding is reasonable and can be explained as follows. It was reported that a 10 to 20 mm leg length discrepancy was the threshold for affecting postoperative gait symmetry and future clinical outcomes (49–51). Lai et al. reported better gait symmetry and efficiency in patients with unilateral Crowe type IV DDH when the leg length discrepancy was within 2 cm (49). Within this range, compensation from the knee and non-operated side was common, which might reduce the potential gait asymmetry to below the level of significance (22). In our study, the maximum length discrepancy was only 10.4 mm, an effect that was likely minor. As a result, no correlation was detected between leg length and gait symmetry. Vertical hip center positions, in turn, became the influential factor for gait symmetry. Interestingly, it was proven from the other side that within the same change of less than 10 mm, the vertical hip center positions had greater power in gait symmetry. This indicates the importance of ensuring appropriate and precise hip center positions during surgery.

The results of this study should be interpreted in light of several limitations. First, the sample size was relatively small, and the results might not be robust. However, considering that unilateral DDH cases are relatively rare and reasonable results were obtained, we still chose to present our findings. This was a pilot study whose results require confirmation in future studies with larger sample sizes. The complexity of the DFIS technique might be another reason for the small sample size in related studies. Second, numerous factors might influence postoperative gait symmetry, including bone, prosthesis, and soft-tissue features. We included a wide range of potential factors including demographics, pre- and postoperative anatomical structures, and surgical changes. The results of the current study were statistically significant. Third, the current study examined only the kinematic variables of the hip joint. In future work, we will attach important kinetic parameters, such as muscle moments and hip-adjacent joint measurements of the pelvis and knee, to comprehensively explore the gait patterns of patients with DDH after THA. Nevertheless, this study showed the critical influence of the postoperative hip center position on gait symmetry and provided potential reconstruction suggestions that could probably inform surgical guidelines.

## Conclusion

The current study first quantified gait symmetry in patients with DDH following unilateral THA during level walking using the DFIS and then explored its potential relationship with demographic information, pre-and postoperative joint structures, and changes in surgery. Our study showed that only 43% of these patients had a symmetrical gait. Postoperative joint structures, especially the hip center positions, significantly contributed to gait symmetry in the frontal and transverse planes. With the hip centers between 17 mm medially and 16 mm laterally, the range of axial rotation was bilaterally symmetrical, which could be a surgical suggestion for surgeons during hip joint reconstruction in THA for patients with DDH.

## Data Availability

The original contributions presented in the study are included in the article/Supplementary Material, further inquiries can be directed to the corresponding authors.
